# Taking Photographs with a Microscope

**DOI:** 10.4269/ajtmh.2009.08-0256

**Published:** 2008-09

**Authors:** Richard J. Maude, Gavin C. K. W. Koh, Kamolrat Silamut

**Affiliations:** Centre for Tropical Diseases, Nuffield Department of Clinical Medicine, John Radcliffe Hospital, Oxford, United Kingdom; Mahidol-Oxford Tropical Medicine Research Unit (MORU), Faculty of Tropical Medicine, Mahidol University, Ratchathewi District, Bangkok, Thailand

## Abstract

We describe a simple, economical, and highly practical technique for taking digital photographs of specimens visualized through a light microscope. Most models of light microscope and compact digital camera, and even some cameraphones, can be used. The technique is quick to learn and can easily be performed in a resource-poor setting. It can be used to assist with diagnosis in remote areas and can be extremely useful for teaching.

Obtaining images of clinical specimens viewed by a microscope can be invaluable for both diagnosis and teaching. Photography by a microscope normally requires a specially adapted microscope with a camera port, a camera with removable lens system, and an adapter to attach the camera to the port.^1^ This equipment is prohibitively expensive for many laboratories, especially in the developing world. The advent of affordable compact digital cameras with displays that reflect precisely the image as seen through the camera lens has made possible a simplified method of taking photographs through a microscope. Almost any combination of light microscope and compact digital camera with optical zoom (including some camera phones) can be used without the need for specialized equipment. Images obtained can be transmitted instantaneously by e-mail or mobile phone picture message for further timely examination by a specialist at another location (e.g., to check species identification of a parasite). This technique therefore has great potential to enhance clinical care in resource poor settings, including in remote areas. It also enables the accumulation of a library of locally relevant clinical images for use in teaching laboratory staff and clinicians and for documentation in research.

The minimum equipment required for this technique is a microscope with eyepiece and a compact digital camera or cameraphone with auto focus, through-the-lens light meter, minimum ×3 optical zoom, and LCD screen and a microscope with eyepiece. The technique is as follows:

1. Using the microscope, examine the specimen by eye and select the area of interest and magnification required.
2. Increase the light source to maximum intensity.
3. Hold the camera lens against the microscope eyepiece. A rubber cup over the eyepiece helps to hold the camera steady. A small circle of light will be seen on the camera's LCD screen.
4. Use the camera's zoom function to increase the size of the circle as required. The most difficult step is moving the camera lens small distances across the eyepiece to center the circle. The camera's autofocus should then self-adjust to give a clear image.
5. Adjust the fine focus of the microscope to maximise image clarity.
6. If the image is too dark or grainy, the camera's ISO setting should be increased (usually 100 or 200 will suffice) or the “darkness” or “night time” setting selected, depending on the model of camera. Note that generally, the higher the ISO, the more difficult it is to obtain a clear image.
7. While holding the camera very still, a photograph can be taken, and the image can be examined to see if it is satisfactory. Excessive blur from camera shake can be minimized using a remote control, by attaching the camera to the microscope using sticky tape or bungee cords or by constructing a frame to hold the camera in place.

Figure 1 shows photographs taken using this technique.

**Figure 1.**
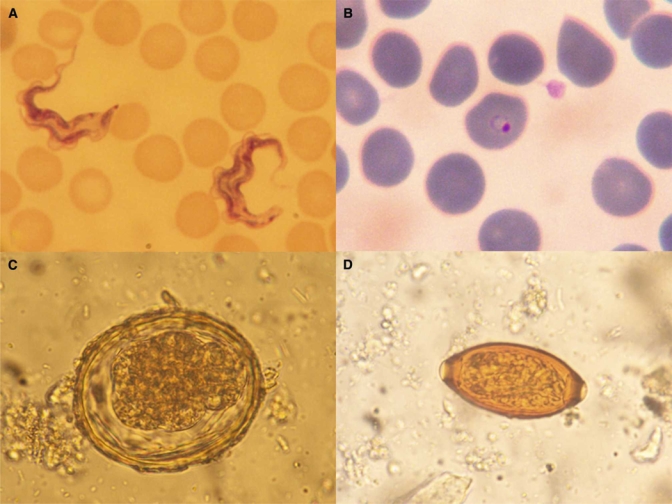
Dividing forms of *T. brucei* (**A**) and ring form of *P. falciparum* (**B**) in peripheral blood film, ×1000 magnification. Corticated *A. lumbricoides* ovum (**C**) and *T. trichiura* ovum (**D**) in stool, ×400 magnification. A variety of staining techniques are shown.
